# Time at Treatment of Severe Retinopathy of Prematurity in China: Recommendations for Guidelines in More Mature Infants

**DOI:** 10.1371/journal.pone.0116669

**Published:** 2015-02-09

**Authors:** Yi Chen, Jing Feng, Clare Gilbert, Hong Yin, Jianhong Liang, Xiaoxin Li

**Affiliations:** 1 Department of Ophthalmology, People’s Hospital, Peking University, & Key Laboratory of Vision Loss and Restoration, Ministry of Education, Beijing, China; 2 International Centre for Eye Health, Department of Clinical Research, London School of Hygiene and Tropical Medicine, London, United Kingdom; University of Sydney, AUSTRALIA

## Abstract

**Purpose:**

To investigate the postmenstrual (PMA) age at treatment of severe retinopathy of prematurity (i.e. Type 1 prethreshold or threshold) in infants in a tertiary referral center in China.

**Principal Findings:**

76.6% (359/469) of infants were treated for threshold disease. 67.5% (317/469) of infants had a birth weight (BW) of 1250g or above and almost 30% (126) had a gestational age (GA) of 32 weeks or above. There was little difference in the characteristics of infants treated for Type 1 prethreshold or threshold ROP. After controlling for GA, PMA age at treatment was highest in infants with BW ≥2000g (mean PMA 40.3±4.4 weeks, p<0.001); after controlling for BW, higher GA was associated with higher PMA at treatment (mean PMA 41.5 weeks for gestational age >34 weeks, p<0.001). For every three weeks increase in GA there was a two-week increase in PMA at treatment (R2 = 0.20, p<0.001). The time at treatment of Type 1 prethreshold disease was similar to that for threshold disease i.e. chronological age 5.6∓7.4 weeks, or PMA 34.1∓40.2 weeks but the lower end of the 95% confidence interval for chronological age for Type 1 prethreshold disease among infants with BW ≥2000g was 3.7 weeks (i.e. before the recommended interval of 4∓6 weeks after birth).

**Significance:**

The Chinese guidelines regarding timing of the first examination are appropriate for infants with BW <2000g, but more mature infants should be examined a little earlier, at 3 weeks after birth, in order to detect Type 1 prethreshold disease which has a better prognosis than threshold.

## Introduction

Retinopathy of prematurity (ROP) is emerging as a major cause of blindness in children in the middle-income countries of Latin American and Asia (China, Thailand, India, and Vietnam) where neonatal intensive care services have improved the survival of high-risk neonates.[1–5] ROP is can be very variable in its severity and outcome. If disease with a high probability of progression to retinal detachment is detected early and treated with peripheral retinal laser, the risk of blindness is reduced.[6,7] However, if the disease progresses to stage 5 ROP the visual acuity prognosis after vitro-retinal surgery is usually poor.[8–10] Thus, timing is an important factor in the detection of ROP requiring laser treatment, as the disease can advance very quickly and delayed treatment reduces the likelihood of a good outcome.

Our previous study in China found that infants who developed severe ROP were more mature and bigger than infants similarly affected in high income countries, and this is reflected in the wider screening criteria being used in China (i.e. birth weight (BW) ≤2000g or gestational age (GA) ≤34 weeks GA) [11–14]. The rate of progression of ROP, and the period after birth during which disease requiring treatment is likely to develop are important as they influence timing of the first and subsequent examinations. [15,16] Data from the control arm of the CRYO-ROP trial indicated that more premature infants developed threshold disease at an earlier postmenstrual age (PMA) than more mature infants, and this is reflected in some country’s national ROP guidelines.[17,18] For example, the US guidelines recommend that infants with a GA of 22 weeks or less be examined at up to 9 weeks after birth which translate to a PMA age of 31 weeks.[13] Infants born at 30 weeks should be examined 4 weeks after birth, which translates to a PMA age of 34 weeks. The UK guidelines are similar [14] However, all the natural history data from the CRYO-ROP trial were on infants with BWs <1251g, being much lower than infants who are currently developing ROP requiring treatment in China.[19]

Indeed, the Chinese guidelines recommend that the initial examination be undertaken 4–6 weeks after birth, or at 32–34 weeks PMA.[12,13] However, these guidelines may not be applicable for the more mature infants who develop severe ROP in China where there is greater exposure to known risk factors that might influence and accelerate the progression of ROP. If more mature infants develop ROP requiring treatment more quickly than those with GA <32 weeks, then some infants may be examined too late in China. There is little information regarding the PMA at which more mature infants are at risk of ROP in middle and low-income countries although a study in Vietnam showed that PMA at threshold in Vietnam was similar to that in developed countries. [20]The aim of this study was to investigate the PMA at treatment of severe ROP among more mature infants compared with less mature infants, to assess whether the Chinese guidelines regarding timing of the first examination need to be modified.

The study was undertaken in the Department of Ophthalmology, People’s Hospital of Peking University, which is the main ROP referral center in northern China. Most units in northern China only screen infants and refer those needing treatment to this hospital as lasers or skills in treatment are not yet available in many units.

## Patients and Methods

The study was conducted in accordance with the Declaration of Helsinki, and we received approval from the Investigational Review Board of the People’s Hospital affiliated with Peking University. Informed consent for all examinations and procedures was obtained from the parents or guardians, who provided their written informed consent to participate.

Our indications for laser treatment in study were eyes Type 1 prethreshold ROP or threshold ROP depending on the stage of disease at presentation.[21] Data were retrieved on all infants treated with laser for Type 1 prethreshold or threshold ROP in the hospital from 2004 to 2011. Medical histories were reviewed and the following data extracted: single or multiple birth, BW, GA, sex, and PMA at treatment (GA + chronological age in weeks). The stage of ROP at the first procedure was recorded. If the baby had different stages of ROP in each eye, the more advanced stage was recorded as the stage for the baby. ROP was classified according to the international classification.[22]

Mean BW and GA were determined for infants treated for Type 1 prethreshold and for threshold ROP. Associations between BW and GA with timing of ROP treatment were determined based on analysis of variance (ANOVA) by considering the effects of BW and GA simultaneously. A commercially available statistical software package (SPSS for Windows version 17.0; SPSS Inc., Chicago, IL) was used for analysis. A one-sample Kolmogorov–Smirnov test was performed to determine whether the samples were normally distributed. Differences between two groups were estimated with a nonparametric Mann–Whitney rank sum test and t test when appropriate. Parameters were compared using the Kruskal–Wallis H test and one-way ANOVA to compare variables among different groups. Two-tailed probabilities less than 0.05 were considered statistically significance.

## Results

A total of 469 infants were treated between 2004 and 2011. 165 were female (35.2%), 68 were twins (14.3%), and one was a triplet (0.01%). The vast majority were treated at the time of diagnosis or within a few days. 76.6% (359) were treated at the threshold level of disease (Table 1). 67.5% (317) of infants had a BW of 1250g or above and almost 30% (126) had a GA of 32 weeks or above.

**Table 1:** Birth weight and gestational age of infants treated for prethreshold and threshold ROP.

	Prethreshold (N = 110, 23.5%)		Threshold (N = 359, 76.5%)		Total (N = 469, 100%)		P value
	Mean ± 1SD	Median (95% CI)	Mean ± 1SD	Median (95% CI)	Mean ± 1SD	Median (95% CI)	
BW (g)	1415.3±347.3	1350.0 (1349.9–1480.6)	1419.4±350.2	1350.0 (1383.0–1455.6)	1418.4±349.2	1350.0 (1386.8–1450.0)	0.86^a^
GA (weeks)	30.3±2.2	30.0 (29.9–30.7)	30.3±2.1	30.0 (30.0–30.5)	30.3±2.1	30.0 (30.1–30.5)	0.69^a^
PMA (weeks)	38.1±3.6	37.0 (37.5–38.8)	38.0±3.8	38.0 (37.6–38.4)	38.0±3.8	37.0 (37.7–38.4)	0.83^a^
CA (weeks)	7.8±3.4	8.0 (7.2–8.5)	7.7±3.5	8.0 (7.4–8.1)	7.7±3.5	8.0 (7.4–8.1)	0.71^a^

BW = birth weight; GA = gestational age; PMA = postmenstrual age; CA = chronological age

^a^ Comparing between prethreshold vs. threshold.

**Figure 1:**
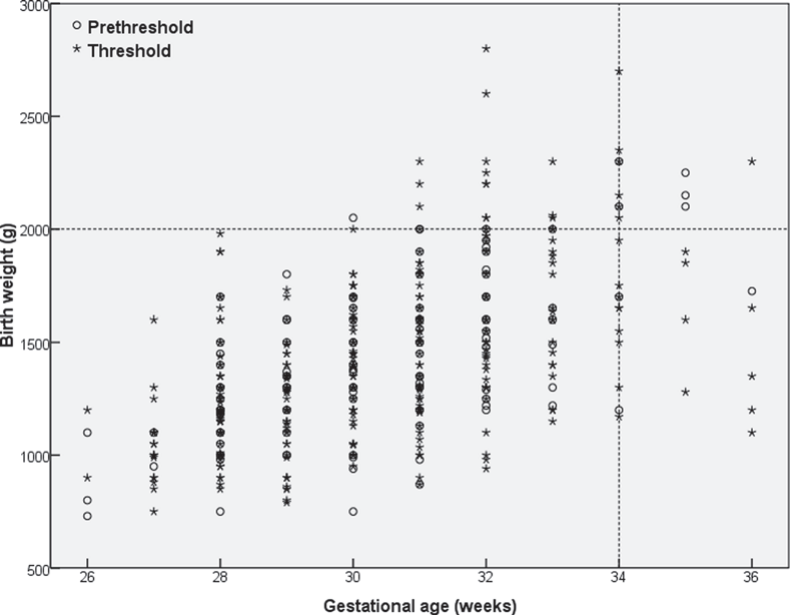
Birth weight against gestational age of 469 infants treated for threshold or prethreshold ROP. Dotted line indicates Chinese screening criteria.

There was little difference in the characteristics of infants treated for Type 1 prethreshold disease or for threshold ROP (Table 1). Four infants (0.8%) had BWs and GAs outside the Chinese screening criteria (≤2000g BW or ≤34 weeks GA) (Fig. 1).

**Figure 2:**
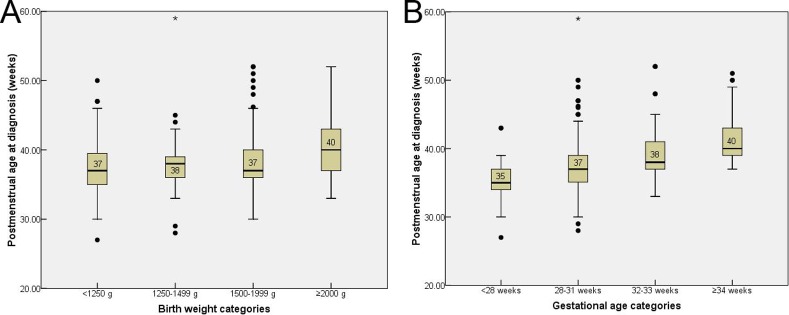
Box plot of postmenstrual age at the time of diagnosis of ROP needing treatment, by birth weight (p = 0.001) (A); Box plot of postmenstrual age at the time of diagnosis of ROP needing treatment, by gestational age (p<0.001) (B).

**Figure 3:**
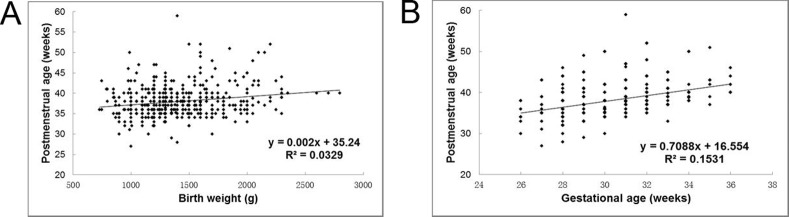
Plot of birth weight against postmenstrual age at treatment (A); Plot of gestational age against postmenstrual age at treatment (B).

Both BW and GA were independently associated with PMA and chronological age at treatment. After controlling for the effect of GA, PMA at treatment was highest in infants with BW ≥2000g (mean PMA 40.3 ± 4.4 weeks, p<0.001) (Table 2, Fig. 2A). After controlling for the effect of BW, higher GA was associated with higher PMA at treatment (mean PMA 41.5 weeks for GA >34 weeks) (p<0.001) (Table 2, Fig. 2B). Linear regression indicated that for every three weeks increase in GA there was only a two-week increase in PMA at treatment (R^2^ = 0.20, p<0.001) (Fig. 3B).

**Table 2:** Postmenstrual and chronological age at treatment, by birth weight and gestational age (all infants).

			Postmenstrual age (weeks)		Chronological age (weeks)	
Birth weight categories	N	%	Mean ± 1SD	Median (95% CI)	Mean ± 1SD	Median (95% CI)
<1250 g	152	32.4%	37.4±3.7	37.0 (36.8–38.0)	8.3±3.5	8.0 (7.8–8.7)
1250–1499 g	137	29.2%	37.5±2.7	38.0 (37.1–38.0)	7.6±2.7	8.0 (7.1–8.0)
1500–1999 g	142	30.3%	38.3±3.8	37.0 (37.7–39.0)	7.2±3.5	7.2 (6.6–7.8)
≥2000 g	38	8.1%	40.3±4.4	40.0 (38.9–41.8)	7.6±4.3	6.9 (6.2–9.1)
Total	469		38.0±3.6	37.0 (37.6–38.3)	7.7±3.4	8.0 (7.4–8.0)
P-value			0.001*		0.001*	
Gestational age categories						
<28 weeks	28	6.0%	35.4±3.3	35 (34.1–36.7)	8.7±3.3	8.0 (7.4–10.0)
28–31 weeks	315	67.2%	37.4±3.5	37 (37.1–37.8)	8.0±3.4	8.0 (7.6–8.3)
32–33 weeks	94	20.0%	39.5±3.7	38 (38.7–40.2)	7.1±3.8	6.0 (6.4–7.9)
≥34 weeks	32	6.8%	41.5±3.7	40 (40.2–42.8)	6.8±3.5	6.0 (5.6–8.0)
Total	469		38.0±3.6	37.0 (37.6–38.3)	7.7±3.4	8.0 (7.4–8.0)
P-value			<0.001*		<0.001*	

* Statistically significant differences at p<0.05

The timing of treatment for Type 1 prethreshold and threshold disease was similar to that for the whole group of infants i.e. 5.6–7.4 weeks chronological age, or 34.1–40.2 weeks PMA (Table 3, Table 4). However, the lower end of the 95% confidence interval for chronological age at treatment of infants with BWs ≥2000g treated for Type 1 prethreshold disease was 3.7 weeks (i.e. before the recommended interval of 4–6 weeks after birth) (Table 3). These infants with BW ≥2000g had a mean gestational age of 33.0 weeks (range: 30.1–35.9 weeks).

**Table 3:** Postmenstrual and chronological age at treatment, by birth weight and gestational age (prethreshold).

			Postmenstrual age (weeks)		Chronological age (weeks)	
Birth weight categories	N	%	Mean±1SD	Median (95%CI)	Mean±1SD,	Median (95%CI)
<1250 g	36	32.7%	37.5±3.9	36.5 (36.2–38.8)	8.5 (3.7)	8.0 (7.3–9.8)
1250–1499 g	29	26.4%	38.6±3.0	38.0 (37.5–39.7)	8.7 (2.9)	8.0 (7.6–9.8)
1500–1999 g	35	31.8%	37.8±3.4	37.0 (36.6–39.0)	6.7 (3.2)	6.0 (5.6–7.8)
≥2000 g	10	9.1%	39.1±3.3	38.5 (36.7–41.5)	6.1 (3.4)	6.5 (3.7–8.5)
Total	110		38.0±3.4	37.0 (37.4–38.7)	7.8 (3.4)	8.0 (7.1–8.4)
P-value			0.158		0.01*	
Gestational age categories						
<28 weeks	6	5.4%	34.8±1.9	34.5 (32.8–36.9)	8.5±2.2	8.0 (6.2–10.8)
28–31 weeks	73	66.4%	37.8±3.5	37.0 (36.9–38.6)	8.3±3.6	8.0 (7.5–9.1)
32–33 weeks	23	20.9%	38.6±2.9	38.0 (37.3–39.9)	6.3±3.0	6.0 (5.0–7.7)
≥34 weeks	8	7.3%	41.1±2.9	41.0 (38.7–43.5)	6.5±2.5,	7.0 (4.4–8.6)
Total	110		38.0 (3.4)	37.0 (37.4–38.7)	7.8±3.4	8.0 (7.1–8.4)
P-value			0.001*		0.092	

* Statistically significant differences at p<0.05

**Table 4:** Postmenstrual and chronological age at treatment, by birth weight and gestational age (Threshold).

			Postmenstrual age (weeks)		Chronological age (weeks)	
Birth weight categories	N	%	Mean±1SD	Median (95%CI)	Mean±1SD	Median (95%CI)
<1250 g	116	32.3%	37.4±3.6	37.0 (36.7–38.0)	8.2±3.4	8.0 (7.6–8.9)
1250–1499 g	108	30.1%	37.2±2.5	37.6 (36.7–37.7)	7.3±2.5	8.0 (6.8–7.7)
1500–1999 g	107	29.8%	38.5±4.0	38.0 (37.7–39.2)	7.3±3.6	6.0 (6.6–8.0)
≥2000 g	28	7.8%	40.8±4.8	40.0 (38.9–42.6)	8.2±4.5	7.4 (6.5–9.9)
Total	359		37.9±3.7	38.0 (37.5–38.3)	7.7±3.4	8.0 (7.3–8.0)
P-value			0.001*		0.019	
Gestational age categories						
<28 weeks	22	6.1%	35.6±3.6	35.0 (34.0–37.2)	8.8±3.6	8.0 (7.2–10.4)
28–31 weeks	241	67.1%	37.3±3.1	37.0 (36.9–37.7)	7.8±3.1	8.0 (7.4–8.2)
32–33 weeks	71	19.8%	39.7±3.9	38.0 (38.8–40.7)	7.4±4.0	6.0(6.4–8.3)
≥34 weeks	25	7.0%	41.4±3.7	40.0 (39.8–42.9)	6.7±3.7	6.0 (5.1–8.2)
Total	359		38.0±3.7	38.0 (37.5–38.3)	7.7±3.4	8.0 (7.3–8.0)
P-value			<0.001*		0.001*	

* Statistically significant differences at p<0.05

## Discussion

With the increased survival of premature infants in China, the incidence of severe ROP is predicted to increase.[12,23] In our previous study, we reported a repeat of the first epidemic of severe ROP from 2001 to 2004 in China, and showed that larger and more mature infants are also at risk.[24] In a more recent study the findings were similar with mean BW and mean GA at treatment being 1418 g and 30 weeks respectively. There may be many reasons for this first epidemic picture in China, but lack of meticulous monitoring of blood oxygen levels, and high mortality rates in very low BW babies are likely explanations.

The criteria for ROP screening in countries with advanced neonatal services are similar, using GA and BW cutoffs based on epidemiologic data. The goal is to ensure that all infants at risk of severe ROP are examined. In this study the vast majority of infants fell within the current Chinese screening guidelines (99.2%), but the wider screening range greatly increases the number of infants to be examined.

Data from CRYO-ROP and ET-ROP studies showed that infants with BWs <1,251g developed threshold ROP at a median PMA of 37 weeks, and Type 1 prethreshold at a slightly earlier PMA (median 36 weeks). [15,25] Our results are similar as infants with BW <1250g developed severe ROP at a median PMA at treatment of 37 weeks. These findings suggest that the progress of the disease is similar across ethnic and racial groups despite exposure to different risk factors. However, none of the earlier studies included more mature infants. Our study of GA categories showed a similar trend, for example those whose GA<28 weeks were treated at a PMA of 35 weeks, and those with GA 32–33 weeks were treated at a PMA of 38 weeks. These findings show that in more mature infants the interval between birth and the onset of severe ROP continues to shorten.

Many factors are involved in the pathogenesis of ROP which are also likely to influence the time of onset and rate of progression.[26] In our study, linear regression indicated that for every three weeks increase in GA there was a two-week increase in PMA at treatment, suggesting that GA is a better predictor of time to development of severe disease than BW, as had been found in other studies. Our study further confirms what is already known about the influence of GA on the timing of the first examination and the time period after birth at which disease requiring treatment is likely to develop, which is a critical factor for ROP programs. [14] However, there is potential for error in the calculation of GA which can be calculated from the start of the last menstrual period, and by ultrasound scanning which is more accurate.[27]However, up to 30% of women have long or irregular cycles [28] and some mothers do not remember the exact date of their last menstrual period.

Based on published data from the CRYO-ROP study, the number of early screening examinations cannot safely be reduced using a single criterion. Using chronological age alone will increase the risk of missing the onset of threshold ROP in larger BW infants while PMA alone will increase the risk in lower BW infants. The data from Hutchinson et al. also suggest that the first examination can often be safely delayed using the combined criteria of chronological and PMA.[29] The current Chinese guidelines recommend that the first examination be at 4–6 weeks after birth, or 32–34 weeks PMA, which is similar to the US and UK guidelines. [11–14] Data from our study showed that the timing of infants with ROP requiring treatment was 5.6–7.4 weeks chronological age and 34.1–40.2 weeks PMA. The ETROP study indicated that early treatment of “high-risk prethreshold ROP” significantly reduced unfavourable outcomes to a clinically important degree.[21] As treatment at Type 1 prethreshold disease has a better prognosis than treatment at threshold, it is desirable that timing of the first examination be set to detect Type 1 prethreshold ROP.[25] In our study the median chronological age at treatment of Type prethreshold disease amongst infants with BW ≥2000g was before the timing of the first examination recommended in the guidelines. In China infants with BW ≥ 2000g should, therefore, be examined at three weeks after birth, which may have the added advantage that the first examination will be undertaken on a higher proportion of infants before discharge.

In our study there were more male than female infants, which probably reflects the preponderance of male children in the population [30] [An additional factor may relate to differences in health seeking behavior between parents of boys compared with girls.

The limitation of our study was the study design, as we performed a retrospective, observation study of data extracted from medical records and databases. Thus, our findings should be interpreted with caution. Ideally, a large, carefully designed, prospective population-based cohort study of ROP should be conducted throughout China.

In conclusion, the recommended timing of the first examination is appropriate for less mature infants with BW <2000g, but bigger infants with BW ≥ 2000g should be examined a little earlier at 3 weeks after birth, to detect Type 1 prethreshold disease when the prognosis is better than at threshold.
